# Traditional Chinese medicine, Danlou tablets alleviate adverse left ventricular remodeling after myocardial infarction: results of a double-blind, randomized, placebo-controlled, pilot study

**DOI:** 10.1186/s12906-016-1406-4

**Published:** 2016-11-08

**Authors:** Shuai Mao, Lei Wang, Wenwei Ouyang, Yuanshen Zhou, Jianyong Qi, Liheng Guo, Minzhou Zhang, Aleksander Hinek

**Affiliations:** 1 Second Clinical Medical College, Key Discipline of Integrated Traditional Chinese and Western Medicine, Guangzhou University of Chinese Medicine, Guangzhou, 510405 China; 2 Department of Critical Care Medicine, Guangdong Provincial Hospital of Chinese Medicine, Guangzhou, 510120 China; 3 Department of Public Health Sciences, Health Systems and Policy, Karolinska Institutet, Stockholm, 17177 Sweden; 4 Physiology & Experimental Medicine Program, Hospital for Sick Children, Toronto, M5G1X8 Canada

**Keywords:** Ventricular remodeling, Myocardial infarction, Danlou tablets, Left ventricular end-diastolic volume index

## Abstract

**Background:**

Danlou tablets, a patented Chinese Medicine, have been long approved for the treatment of ischemic heart disease in China. While numerous empirical observations suggested Danlou tablets could decrease frequency and duration of angina pectoris attacks, evidence supporting its efficacy on cardiac remodeling remains inadequate. Therefore, this pilot trial was designed to determine whether Danlou tablets would reduce adverse left ventricular (LV) remodeling in patients with myocardial infarction (MI).

**Methods and Results:**

Eligible patients with acute MI were enrolled and randomly assigned to Danlou tablets or placebo groups, superimposed on standard treatment for MI. Then, in addition to assessment of the clinical outcome, the changes in LV volumes were evaluated by a serial echocardiography. In total, 83 patients (Danlou tablets 42 and placebo 41) completed 90 days of treatment and had complete baseline and outcome data. Standard echocardiographic evaluations revealed significant differences in the change of LV end-diastolic volume index (LVEDVi) between group of patients treated with Danlou tablets and the placebo group (−4.49 ± 7.29 vs. −0.34 ± 9.01 mL/m^2^, *P* < 0.001). The reduction in LVEDVi was independent of beta-blocker, ACE inhibitors/ARBs use. Furthermore, treatment with Danlou tablets significantly reduced LV end-systolic volume index (−4.09 ± 5.85 vs. −0.54 ± 5.72 mL/m^2^, *P* < 0.001) and improved the LV ejection fraction (4.83 ± 9.23 vs. 0.23 ± 8.15 %, *P* < 0.001), as compared to placebo. Meaningfully, the incidence of the major adverse cardiovascular events was also lower in patients receiving Danlou tablets (*P* < 0.05).

**Conclusion:**

Superimposed on the standard pharmacologic treatment, Danlou tablets significantly reversed post-MI adverse LV remodeling, thereby contributed to the overall positive clinical outcome.

**Trial registration:**

clinicaltrials.gov identifier NCT02675322 (February 1, 2016).

**Electronic supplementary material:**

The online version of this article (doi:10.1186/s12906-016-1406-4) contains supplementary material, which is available to authorized users.

## Background

Adverse left ventricular (LV) remodeling after myocardial infarction (MI) has been shown to be associated with an increased risk for adverse cardiovascular events, whereas the progressive deterioration of cardiac performance is an important warning sign of the developing heart failure and a significant predictor of all-cause mortality [1–3]. To date, several therapeutic approaches have been exercised in patients afflicted with acute MI, including local coronary reperfusion and systemic treatments with inhibitors of angiotensin converting enzyme (ACE), beta-adrenergic blockers, as well as application of pharmacologic or electrical cardiac resynchronization [4–7]. While numerous clinical trials already proven that these methods prevent development of the LV dilatation to a certain degree, the incidences of the adverse LV remodeling, still observed in a substantial proportion of patients after MI, remains the major challenge for modern medicine [8, 9]. Therefore, there is a constant need for development of novel and more efficient therapies that would reverse LV remodeling and reduce the incidence of major cardiac events.

Over the past few years, numerous components of the traditional Chinese Medicine (TCM) that would exert cardioprotective effects, received a great deal of attention and have been tested for their abilities in alleviating symptoms of angina pectoris, myocardium infarction, heart failure, arrhythmia, and other cardiovascular conditions [10–14]. In particular, Danlou tablets (consisting of *Salvia miltiorrhiza, Ligusticum chuanxiong Hort, Trichosanthes kirilowii, Allium macrostemon Bung, Radix Paeoniae Rubra, Radix Puerariae, rhizoma alismatis, Curcuma aromatic, Rhizoma Drynariae, and Astragalus membranaceus*) known for their anti-oxidant and anti-inflammatory action, have been successfully used by TCM practitioners, for stabilizing atherosclerotic plaques and reduction of the ischemia reperfusion injury [15–17]. From the perspective of TCM, the negative outcome of MI is mostly caused by accumulation of intracardiac “phlegm” and obstruction of coronary circulation. It has been also suggested that treatments of patients with Danlou tablets may contribute to elimination of “phlegm” and restoration of inadequate blood circulation in the afflicted hearts [18, 19]. Therefore, the pilot clinical trial was designed to test the hypothesis that the administration of Danlou tablets would attenuate progressive LV dilatation after acute MI.

## Methods

### Patient population

Patients included in our study were aged 18 to 80 years with typical chest pain lasting for at least 30 min and a subsequent rise in specific cardiac biomarkers of myocardial injury [creatine kinase-MB (CKMB) or troponin T (TnT)] to more than 3 times the normal upper limit, and admitted with acute MI who successfully underwent revascularization (percutaneous coronary intervention, PCI) of the infarct-related artery. Eligible patients also met the diagnostic criteria of intermingled phlegm and blood stasis syndrome according to the criteria for TCM syndrome differentiation of patients with coronary heart disease, published by the Chinese Interactive Medicine Association.

Patients with previous MI within 30 days, malignant arrhythmia, congenital heart disease, cardiac shock, documented or suspected history of heart failure or depressed LV ejection fraction <15 %, a planned coronary artery bypass grafting, a life expectancy of <1 year, hepatic impairment, estimated glomerular filtration rate ≤30 mL/min per 1.73 m^2^, autoimmune or connective tissue disease, chronic substance abuse or psychiatric illness, and who were unable to complete the 3-month clinical follow-up were excluded in this study.

### Study Design and Protocol

This randomized, prospective, double-blind, placebo-controlled, parallel-group clinical trial was performed in two institutions (Guangdong Provincial Hospital of CM and Wuyi Hospital of CM). The study was conducted in accordance with the Declaration of Helsinki and its text revisions, and all participants provided written informed consent before enrollment. The Clinical Research Ethical Committee at Guangdong Provincial Hospital of CM approved the research protocol (B2011-41-01). This trial was registered in clinicaltrials.gov (NCT02675322).

Once eligible candidates had undergone PCI, they were admitted to the coronary intensive care unit and received standard treatment according to the institutional protocol based on American College of Cardiology/ American Heart Association and European Society of Cardiology guidelines [20]. Simultaneously, patients were randomly assigned in a 1:1 ratio to receive Danlou tablets (4.5 g oral dose taken once daily) or matching placebo for 90 days, according to a computer-generated site-stratified, block randomization schedule. Danlou tablets were approved by China Food and Drug Administration for the treatment of ischemic heart disease in 2005 (Z20050244). Blinding will be ensured using a matched placebo granule identical in color, size, shape, and taste. The quality of the matched trial supplies, such as contents, solubility, and bacteria contaminations, should be controlled rigorously according to the good manufacturing practice (GMP) standards, and be tested and verified by researchers.

Circulating levels of CK-MB, TnT, and N-terminal pro-brain natriuretic peptide (NT-proBNP) were systematically measured at admission time. Baseline blood test data were collected and performed by an independent core laboratory blinded to treatment assignment. In addition, two-dimensional Doppler echocardiography was performed within 48 ± 24 h after PCI. Moreover, clinical and echocardiographic follow-up assessments were scheduled in all study patients at 90 days after MI. Clinical events were recorded by investigators from enrollment through 90 days, whereas vital status and adverse events were collected 6 months after assignment.

The primary endpoint of the study was the change in the LVEDVi (ΔLVEDVi), as measured by two-dimensional (2D) echocardiography from baseline to 90 days. Secondary study endpoints were changes in LVESVi (ΔLVESVi) and LVEF (ΔLVEF) during the same interval. In addition, the occurrence and composite of major adverse cardiovascular events (MACE), in terms of cardiac death, recurrent MI, and target vessel revascularization, cardiogenic shock, severe heart failure (hospital readmission for congestive heart failure and/or worsening NYHA class III-IV) and major arrhythmia were assessed and recorded during hospitalization and up to 90 days thereafter. Additional endpoints included the assessment in safety outcomes during the period of follow-up (mean 6 months).

### Echocardiographic imaging and analyses

Echocardiograms were performed by experienced sonographers using a commercially available system (Philips Medical Systems, Andover, MA, USA) for LV dimension and ejection fraction quantification. Standard 2D images and Doppler data were obtained using the standard apical 2- and 4- chamber views, according to the recommendations of the American Society of Echocardiography [21]. The echocardiograms were analyzed centrally, according to predefined procedures in the core laboratory at the Guangdong Provincial Heart Center by technicians supervised by two cardiologists, all of them blinded to the randomized assignment and examination sequence (baseline vs. follow-up assessment). The LV long axis was measured in the apical 2- and 4-chamber views from the apex tip to the anterior/lateral corner of the mitral annulus. Biplane end diastolic and systolic volumes were calculated using the modified Simpson’s rule. Three cycles were measured for each assessment, and the average volumes were obtained. The LVEDVi and LVESVi were then calculated as LVEDV and LVESV divided by body surface area. The LV ejection fraction was calculated using the multiple diameter method, which uses the average of the LV diameters measured at end-diastole and end-systole from the parasternal long axis and apical views together along with an estimate of fractional long axis shortening. Pulsed-wave Doppler of transmitral flow was used to assess global diastolic function, by placing the sample volume between the tips of the mitral leaflets, peak early velocity (E), peak atrial velocity (A), and E-wave deceleration time (EDT) were measured [22]. Internal review of the first 60 paired echocardiograms (baseline and day 90) was performed and verified (<10 % interobserver variability in LV dimension and ejection fraction measurements).

### Statistical analysis

To calculate the target sample size for the present study, we hypothesized that Danlou tablets would decrease the LVEDVi by 8 %. An estimated sample size of 80 patients (40 per group) would be needed to detect the difference with a statistical power 90 % and a probability type I error of 5 %, considering an attrition rate of 10 %. Baseline characteristics were presented with mean ± standard deviation (SD) or median and interquartile range (IQR) for continuous variables, and with numbers and percentages (%) for categorical variables. The analysis would be performed based on the intent-to-treat (ITT) principle. Continuous variables were compared within each group using Student’s *t*-test or the Wilcoxon rank-sum test (if non-normally distributed), whereas the categorical variables were compared using chi-square statistics or Fisher’s exact test when appropriate. To compare the means of basal echocardiography data between groups, two-sided Student’s t-tests were used. 90-day changes from baseline in LVEDVi, LVESVi and LVEF were compared between groups using analysis of covariance with major determinants, including age, concomitant systemic hypertension, diabetes mellitus, peak CK-MB, peak NT-proBNP levels, treatment with beta adrenergic blockers and ACE inhibitors (or ARB), and corresponding baseline echocardiogaphic values that could explain the LV remodeling as covariates. The treatment effect of Danlou tablets (mean difference in change of LVEDVi, LVESVi and LVEF) was estimated using adjusted least square means from this model, with associated two-sided 95 % confidence intervals (CI) and *P*-values (significance level set at 5 %). Statistical analysis was performed using SPSS version 13.0 software (SPSS, Inc., Armonk, NY, USA).

## Results

### Clinical characteristics

**Fig. 1:**
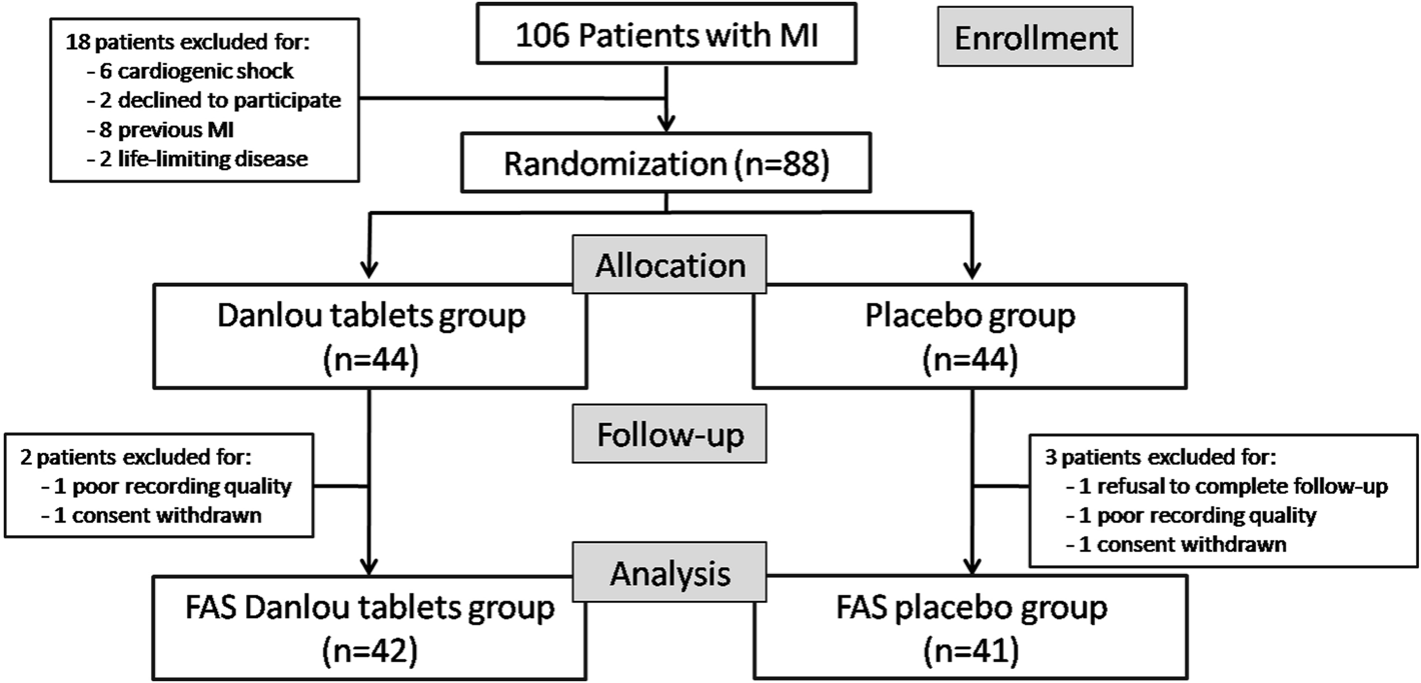
Trial profile. The number of patients who were enrolled in the study and included in the analyses. MI, myocardial infarction; FAS, full analysis set

In total, 106 patients with MI were screened for the study, and initially 88 were included (44 Danlou tablets and 44 placebo; Fig. 1). Five patients were excluded, leaving 83 patients (94.3 % of the initial set, 42 Danlou tablets and 41 placebo) for analysis. The reasons for exclusion included the poor quality of echocardiographic imaging (insufficient resolution of LV endocardial border), no follow-up recording and consent withdrawal. The clinical characteristics of the study population at baseline are shown in Table 1.

**Table 1:** Baseline characteristics data

	Danlou tablets (*n* = 44)	Placebo (*n* = 44)	*P*
Age, yr	67.54 ± 8.39	68.38 ± 10.41	0.18
Male sex, no. (%)	28 (63.64)	27 (61.36)	0.83
BMI, kg/m^2^	22.29 ± 4.78	23.99 ± 3.12	0.35
Heart rate (beats/min)	75.41 ± 13.69	78.84 ± 13.36	0.57
Systolic blood pressure (mmHg)	126.44 ± 26.43	123.42 ± 27.18	0.85
Concomitant diseases, no. (%)			
Previous coronary heart disease	12 (27.27)	15 (34.09)	0.49
Systemic hypertension	21 (47.73)	23 (52.27)	0.67
Diabetes mellitus	9 (20.45)	10 (22.73)	0.80
Dyslipidemia	12 (27.27)	18 (40.91)	0.18
Current smoker, no.(%)	14 (31.82)	12 (27.27)	0.64
NYHA Class			
Class I	4 (9.09)	5 (11.36)	0.99
Class II	20 (45.46)	19 (43.18)	
Class III	18 (40.91)	17 (38.64)	
Class IV	2 (4.54)	3 (6.82)	
Procedural Features			
LAD (%)	35 (79.55)	32 (72.73)	0.45
Multivessel disease (%)	14 (31.82)	19 (43.18)	0.39
Procedural success (%)	44 (100)	44 (100)	1.00
Stenting of IRA (%)	44 (100)	44 (100)	1.00
Multiple stenting (%)	10 (22.73)	8 (18.18)	0.60
Total length of stenting (mm)	20.52 ± 7.01	22.37 ± 6.46	0.22
TIMI flow grade 3 after PCI (%)	44 (100)	44 (100)	1.00
Procedural time (min)	62.68 ± 19.72	65.32 ± 19.55	0.51
Cardiac biomarker			
Peak CK-MB (IU/L)	68.01 [46.83–99.90]	64.34 [19.65–97.98]	0.21
NT-proBNP (pg/mL)	1419.27 [293.41–2678.54]	1147.21 [240.50–2675.61]	0.55
Therapy, no. (%)			
Aspirin	42 (95.45)	43 (97.73)	0.56
Clopidogrel	44 (100)	44 (100)	1.00
Statin	44 (100)	44 (100)	1.00
Beta adrenergic blockers	40 (90.91)	41 (93.18)	0.69
ACE inhibitors (or ARB)	41 (93.18)	42 (95.45)	0.65
Aldosterone antagonist	5 (11.36)	4 (9.09)	0.73
Digitalis	8 (18.18)	6 (13.64)	0.56

Data are presented as mean ± SD or median [interquartile range] or numbers (%) when appropriate

*ACE* denotes angiotensin converting enzyme, *ARB* angiotensin II receptor blocker, *BMI* body mass index, *CK-MB* creatine kinase-MB, *IRA* infarct-related artery, *LAD* left anterior descending artery, *LVEF* left ventricular ejection fraction, N-terminal pro-brain natriuretic peptide, *NYHA* New York Heart Association, *PCI* percutaneous coronary intervention, *TIMI* thromobolysis in myocardial infarction

Study groups were well matched overall without clinically relevant differences in demographic variables, cardiovascular risk factors, concomitant diseases, clinical presentation, or discharge medical therapy. The mean age of the population was 67.97 ± 9.41 years. The majority of patients were men (62.50 %). Mean resting heart rate at baseline was 76.13 ± 13.15 bpm. Comparing the main procedural features in both groups, there were no statistically significant differences in multivessel lesion type, procedural characteristics of intervention, number of stents per patient, and diameter and length of implanted stents (*P* > 0.05, respectively). The patients were evenly distributed between New York Heart Association (NYHA) function classes II to III, along with similar baseline NT-proBNP levels [1419.27 pg/mL (IQR, 293.41 to 2678.54) vs. 1147.21 pg/mL (IQR, 240.50 to 2675.61), *P* = 0.55]. Patients in both treatment groups had high rates (exceeding 90 %) of beta-blocker and rennin angiotensin aldosterone system (RAAS) antagonist use.

### Echocardiographic findings

**Fig. 2:**
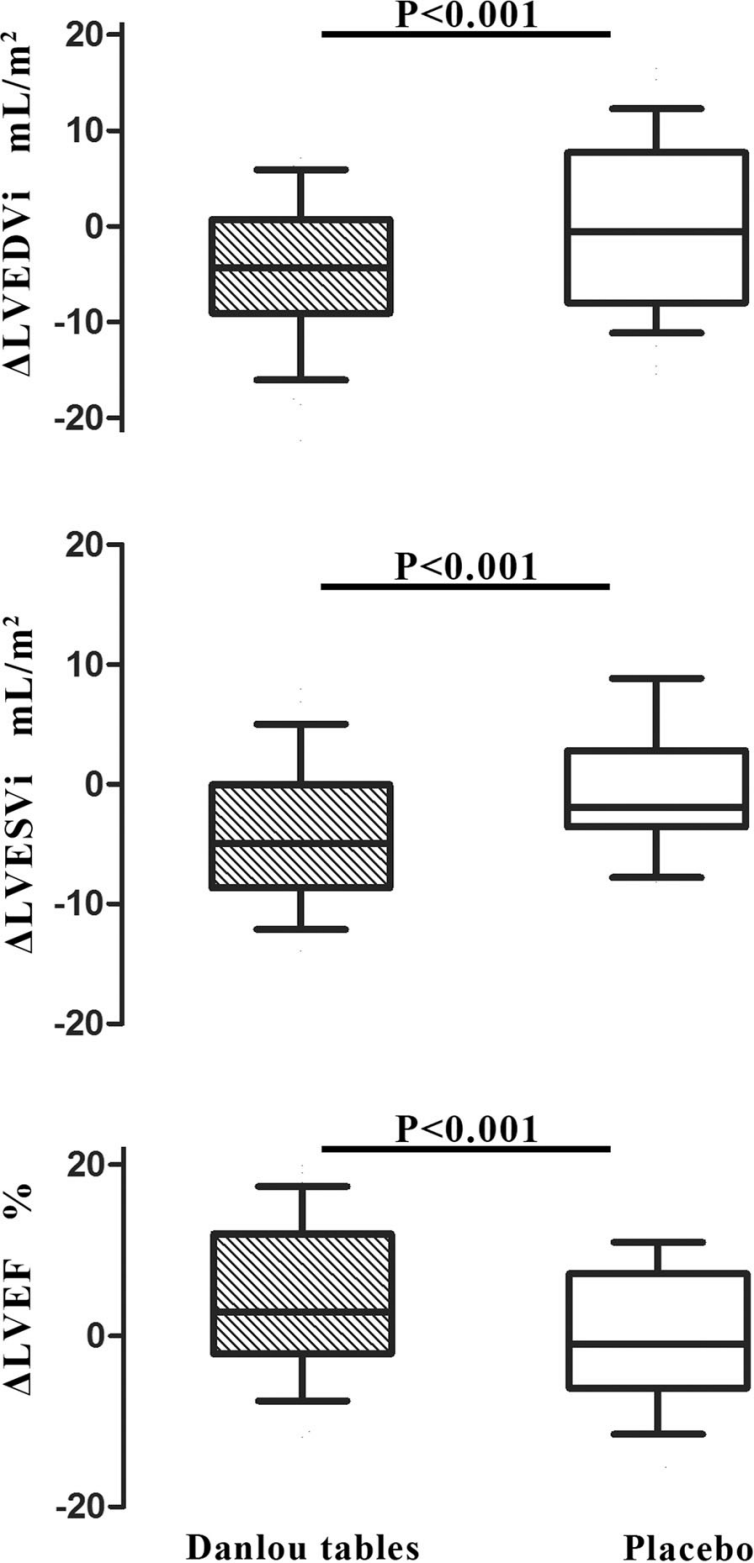
Change in LVEDVi, LVESVi and LVEF from baseline to 90 days. Echocardiographic changes from baseline to 6 months in LVEDVi, LVESVi and LVEF (ΔLVEDVi, ΔLVESVi, ΔLVEF). Middle hash of the box indicates the median; 25 to 75 th percentiles are represented by end caps of the box; the whiskers indicate the 10 and 90 th percentiles. LVEDVi, left ventricular end-diastolic volume index; LVESVi, left ventricular end-systolic volume index; LVEF, left ventricular ejection fraction

Table 2 and Fig. 2 present echocardiographic LV remodeling results from baseline to 90 days. Baseline LVEDVi was similar in the Danlou tablets and placebo groups (49.22 ± 8.70 vs. 51.33 ± 9.61 mL/m^2^, respectively, *P* > 0.05). Treatment with Danlou tablets for 90 days was associated with a significant reduction in LVDSVi as compared with the placebo (−4.49 ± 7.29 vs. −0.34 ± 9.01 mL/m^2^, mean difference −5.86, 95 % CI −7.55 to −4.16, *P* < 0.001).

**Table 2:** Echocardiogaphic LV remodeling end points

	Danlou tablets (*n* = 42)			Placebo (*n* = 41)			Danlou tablets vs. Placebo	*P*
	Baseline	90 days	Change	Baseline	90 days	Change	Difference in change (95 % CI)	
LVEDVi, mL/m^2^	49.22 ± 8.70	44.73 ± 4.29	−4.49 ± 7.29	51.33 ± 9.61	50.99 ± 3.55	−0.34 ± 9.01	−5.86 (−7.55 to −4.16)	<0.001
LEVSVi, mL/m^2^	31.79 ± 5.29	27.69 ± 2.46	−4.09 ± 5.85	31.05 ± 6.02	30.51 ± 3.12	−0.54 ± 5.72	−3.20 (−4.43 to −1.98)	<0.001
LVEF,%	44.76 ± 7.95	49.59 ± 3.29	4.83 ± 9.23	46.79 ± 8.03	46.53 ± 2.97	0.23 ± 8.15	3.15 (1.66 to 4.63)	<0.001

Values are expressed as means ± SD when appropriate

*CI* confidence interval, *LVEDVi* left ventricular end-diastolic volume index, *LVESVi* left ventricular end-systolic volume index, *LVEF* left ventricular ejection fraction

The values of the LVESVi were similar at baseline in the Danlou tablets and placebo groups (31.79 ± 5.29 vs. 31.05 ± 6.02, *P* > 0.05). From baseline to 90-day follow-up, LVESVi remained substantially unchanged in the control group, whereas it was significantly decreased in the Danlou tablets group (−0.54 ± 5.72 vs. −4.09 ± 5.85 mL/m^2^, mean difference −3.20, 95 % CI −4.43 to −1.98, *P* < 0.001).

Consequently, the increase from baseline to the 90-day follow-up of LVEF was higher in the Danlou tablets group than in the control group (4.83 ± 9.23 and 0.23 ± 8.15 %, mean difference 3.15, 95 % CI 1.66 to 4.63, *P* <0.001).

### Clinical follow-up

The adherence of patients to therapy was fully respected. Overall, no patients in either Danlou tablets or placebo group died during their hospital stay and during the follow-up periods. However, 9 patient (21.95 %) from the placebo group and 5 patients (11.90 %) treated with Danlou tablets had a non-fatal recurrent MI. Also, 1 patient (out of 42) from the Danlou tablets group (2.38 %) and 5 patients (out of 41) in the placebo group (12.20 %) developed a severe heart failure. Moreover, 2 patients from the placebo (4.88 %) demonstrated symptoms of the cardiogenic shock and 1 patient (2.44 %) had a major arrhythmia. Importantly, these complications did not developed in patients receiving Danlou tablets. In total, the incidence of composite of reinfarction, severe heart failure, cardiogenic shock and arrhythmia was higher in the placebo group than in the Danlou tablets group (11.90 vs. 34.15 %, *P* = 0.02).

Finally, we like to state that all patients were closely monitored by a set of drug safety analyses. Results of their statistical evaluation indicated that apart of occasional dyspepsia symptoms occurring in Danlou tablet-treated patients, we did not detect any serious adverse events related to our trial during the follow-up period (data not shown).

## Discussion

Adverse LV remodeling commonly occurs after MI, despite the successful coronary reperfusion and application of several classic pharmacological interventions. It often causes a progressive LV dilation that compromises the overall myocardial contractility and eventually culminates in heart failure [23]. Therefore, recent attempts, including the present study, have been aimed at limiting the pathological progress of LV remodeling by administration of certain natural agents derived from complementary and alternative medicine [24].

Results of the double-blind, randomized and placebo-controlled clinical trial demonstrated that treatment with Danlou tablets (combined with standard pharmacological agents) significantly reduced rates of adverse LV remodeling (ΔLVEDVi and ΔLVESVi) and improved the overall clinical outcomes in patients afflicted with acute MI.

Danlou Tablets consist of 11 types of herbs, all of which are included in the Chinese pharmacopoeia. UPLC-MS/MS was also used to analyse 15 quality-control markers of Danlou Tablet, and good consistency of the active markers was found among 8 different batches, including tanshinone IIA, danshen su, puerarin, daidzin, salvianolic acid B, and salvianolic acid A (Additional file 1). The most important determinants characterizing the magnitude and kind of LV remodeling are the actual size of the infarct, the existence of microcirculation in the ischemic zone, as well as the quality and quantity of the newly deposited extracellular matrix (ECM). The Danlou tablet-related improvements of LV remodeling are probably due to its established multiple effects which play vital roles in reversing the pathological remodeling process. Previously published studies of experimental infarcts in animal models suggested that Danlou tablets may protect vascular endothelium and improve formation of new capillaries on the edges of the ischemic myocardial zones, thereby contributed to the ultimate reduction of the infarct size, promotion of myocardial healing and subsequent alleviation of the progressive ventricular fibrosis [25–27]. Our previous studies performed on cultured human cardiac fibroblasts further confirmed that tanshinone IIA, an effective component of Danlou tablets, significantly inhibited the deposition of collagen fibers which exacerbate mechanical stiffness and reduced contractility, but simultaneously enhanced production of new elastic fibers contributing to more resilient matrix [28, 29]. Since the optimal proportions of these ECM components eventually determine the beneficial healing of the infarcted myocardium, we speculate that addition of the Danlou tablets to the classic Western pharmacological therapy of our MI patients, likely shifted the initial adverse remodeling processes toward the ultimate regeneration and formation of the more elastic post-infarct scars, complying with the action of the initially injured hearts and preserving global contractility and compliance. Moreover, results of only few clinical reports also suggested that administration of Danlou tablets in patients with ischemic heart disease induced improvement of clinical angina symptoms and decreased frequency of major atherothrombotic complications (plaque rupture and thrombosis) that eventually lead to MI [30]. However, up to date the observed beneficial effects of Danlou tablets have not been mechanistically confirmed by the adequate randomized, prospective, double-blind, placebo-controlled clinical trial.

The 90-day long duration of this pilot study was encouraged by previous clinical studies indicating that this time is always sufficient for the objective echocardiographic assessment of the changes in LVEDVi [31]. Therefore, we used echocardiography for a standard evaluation of LV remodeling, because this readily available technique permitted for the quick and efficient screening of potential patients, and allowed us for a fair estimation of the benefits of ACE inhibitors and beta-blockers on LV volumes and ejection fractions [32, 33]. During the serial LV remodeling measurements, we detected some individual variability, but its range was rather similar in both tested groups and provided us with an adequate statistical power to revaluate the variation.

Several limitations in this study should be acknowledged. Despite the fact that the indirect measures of myocardial enzyme (peak CK-MB) levels, ventricular wall stress (peak pro-BNP) and myocardial reperfusion effectiveness (TIMI flow grade 3 after PCI) did not significantly differ between both experimental groups (Table 1), we are aware that without quantitative assessment of infarct size and myocardial viability at baseline, a potential selection bias favoring the Danlou tablet therapy arm could not be entirely excluded. While the prognostic value of LVEF assessed early after MI has been previously demonstrated by other studies, we are conscious that results of echocardiography, performed early (within 48 h) after MI, might underestimate the LVEF due to stunning of the myocardium [34]. Despite the above mentioned limitations of our pilot trial, we hope that further studies testing the larger populations will ultimately justify the beneficial impact of Danlou tablets on the post-MI adverse LV remodeling.

## Conclusion

Results of our preliminary clinical trial demonstrated that LVEDVi and LVESVi were significantly smaller in MI patients who received Danlou tablets, as compared to their placebo-treated counterparts. They strongly indicated that addition of this TCM remedy to the standard reperfusion and conventional pharmacologic medical intervention, should be considered as a promising therapeutic approach, aimed at alleviation of the adverse LV remodeling and preserving cardiac function in patients afflicted with acute MI. Therefore, encouraged by the up-today results, we will continue and extend the ongoing clinical trial after randomly assigning 280 more patients with the angiography-confirmed acute MI that could be followed up to 6 months.

## Additional file

Additional file 1: UPLC fingerprints of Danlou Tablets. (TIF 732 kb)

## Abbreviations

ACE: Angiotensin converting enzyme
EF: Ejection fraction
LV: Left ventricular
LVEDV: Left ventricular end-diastolic volume
LVESV: Left ventricular end-systolic volume
MACE: Major adverse cardiac event
MI: Myocardial infarction
NT-proBNP: N-terminal pro-brain natriuretic peptide
NYHA: New York Heart association functional classification
PCI: Percutaneous coronary intervention
TVR: Target vessel revascularization
