# Supplementary figures and images for: Traditional Chinese medicine, Danlou tablets alleviate adverse left ventricular remodeling after myocardial infarction: results of a double-blind, randomized, placebo-controlled, pilot study
Source: BMC Complement Altern Med. 2016 Nov 8;16:447. doi: 10.1186/s12906-016-1406-4 (PMC5101662; doi:10.1186/s12906-016-1406-4)

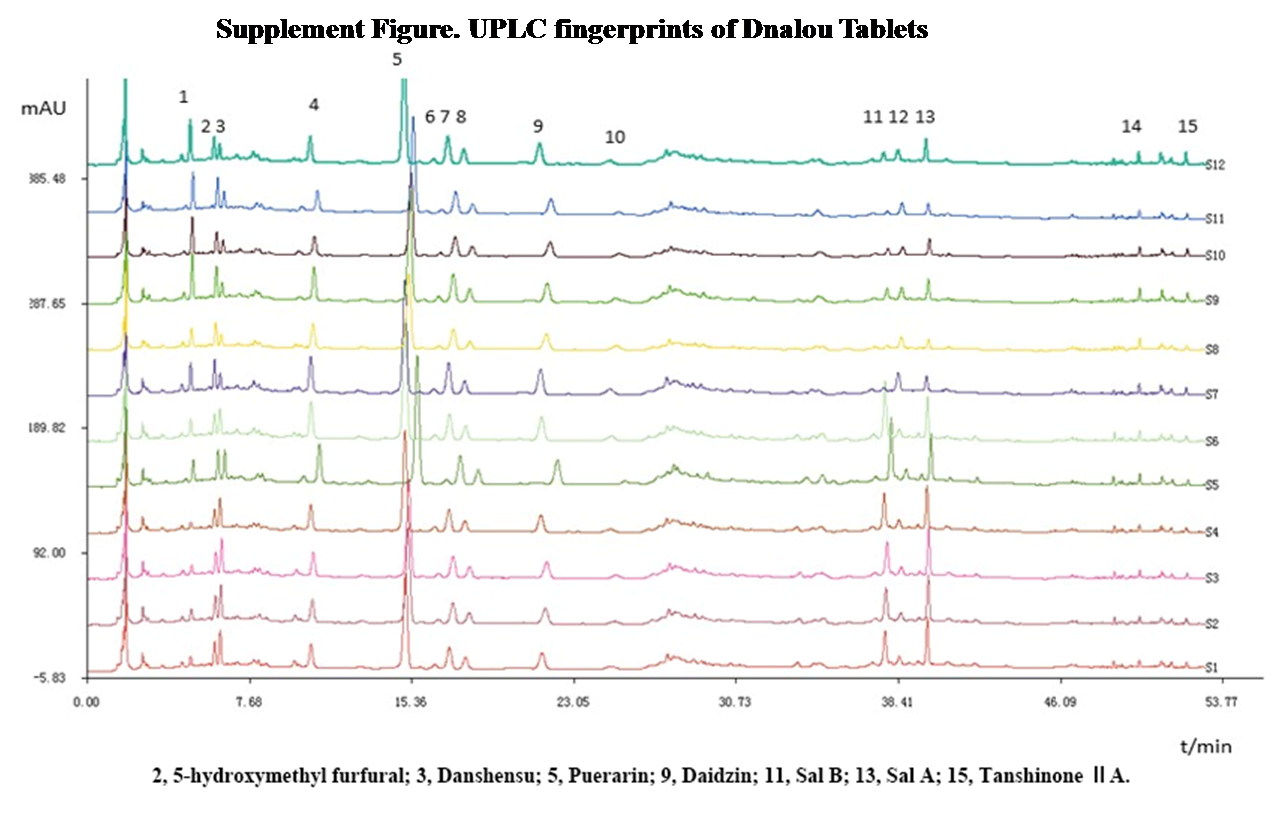

Supplement: Additional file 1: — UPLC fingerprints of Danlou Tablets. (TIF 732 kb) [file 12906_2016_1406_MOESM1_ESM.tif]
